# White Collar Proteins Help Fungi Do It in the Dark

**DOI:** 10.1371/journal.pbio.0030142

**Published:** 2005-03-15

Fungi live mainly in the dark, and they like it that way—out of the sunlight, they can avoid desiccation and damage from ultraviolet rays. The ability to sense light, therefore, is adaptive for fungi of all kinds. A pair of light-sensing proteins had been identified in the model fungus Neurospora crassa, an ascomycete (one of the three fungal subgroups, defined by the production of sexual spores within sac-like structures), but little was known about mechanisms in other fungal phyla. In a new study, Alexander Idnurm and Joseph Heitman show that the basidiomycete (a subgroup defined by the production of sexual spores on the ends of club-like structures) Cryptococcus neoformans employs a similar protein pair, which regulate mating, growth, and virulence of this human fungal pathogen.

In N. crassa, blue light is sensed by the protein White collar 1, which interacts with a flavin (light-absorbing pigment) tuned to photons in the blue region of the spectrum. White collar 1 then binds to White collar 2, and the complex serves as a transcription factor. In this study, the authors searched the C. neoformans genome for genes with similar evolutionary origins to these two genes (called homologs), as well as others implicated in light sensing, and identified *Basidiomycete white collar 1*, or *BWC1*, along with other light-sensor candidates, including an opsin and a phytochrome homolog. Mutations of *BWC1*, but not the other candidate photoreceptors, rendered C. neoformans insensitive to light. While mating and fruiting in the wild-type fungus is suppressed by exposure to blue light, *bwc1* mutants were unaffected by light. Interestingly, the mating process was released from light inhibition when either one of the two mating strains were mutated, suggesting that the cell fusion process at the heart of fungal mating requires only one cell to commit to fusion. In addition, *bwc1* mutants were extremely sensitive to ultraviolet radiation. No homolog of photolyase, a protein that uses light to repair DNA damage, was identified in the genome. Future studies will be necessary to understand how Bwc1 functions in ultraviolet resistance, but these findings suggest the protein could sense photons in both the ultraviolet and blue wavelengths.

To identify other proteins with which the Bwc1 protein functionally interacts, the authors examined nearly 3,000 mutant strains, yielding three with a phenotype similar to the *bwc1* mutant. They found that the gene for one of these, dubbed *BWC2*, is a homolog of N. crassa White collar 2, and that its protein binds to Bwc1. Together, the two influence transcript levels of two key genes required for C. neoformans mating, further strengthening the case that the pair function as a transcription factor as do their homologs in N. crassa. Interestingly, mutants of either *BWC1* or *BWC2* were less virulent than the wild-type strain of the fungus, revealing a novel environmental signaling pathway involved in C. neoformans virulence.

The functional and structural similarities of the ascomycote and basidiomycote White collar proteins indicate that they arose prior to the split of these two lineages more than 500 million years ago. The fungal kingdom contains an estimated one million species. The authors suggest that the ultraviolet protection afforded by the White collar system may have been crucial to the evolutionary diversification of this kingdom, in particular when ultraviolet radiation on the earth's surface was higher than it is today, such as when life emerged from the sea and colonized the barren continents. They also note that the same proteins are found in clinical isolates of C. neoformans, and that the mitigation of virulence by *bwc1* and *bwc2* mutations will be useful in the identification of new genes required for disease development in this important pathogen.

